# Recent advances in the role of CARM1 in skeletal muscle development, metabolism, and homeostasis maintenance

**DOI:** 10.3389/fcell.2025.1709515

**Published:** 2025-12-19

**Authors:** Xiaojing Xie, Menghuan Li, Yue Zhang, Zhenwei Bao, Xuejie Yi

**Affiliations:** 1 School of Sports and Health, Shenyang Sport University, Shenyang, China; 2 School of Physical Education, Liaoning Normal University, Dalian, China; 3 Sports and Health Research Center, Shenyang Sport University, Shenyang, China

**Keywords:** autophagy, CARM1, exercise, metabolism, muscle atrophy, muscle development, oxidative stress, skeletal muscle

## Abstract

The development, metabolism, and functional maintenance of skeletal muscle is a complex dynamic balance process. Its imbalance may lead to muscular dystrophy, muscle atrophy, and other diseases, which seriously affect human health. Therefore, in-depth exploration of the regulatory mechanisms governing skeletal muscle homeostasis and the identification of effective therapeutic targets have garnered significant attention. Recent studies reveal that the protein arginine methyltransferase *CARM1* plays a central regulatory role in skeletal muscle biology. Substantial evidence indicates that abnormal *CARM1* expression and activity disrupt muscle regeneration, metabolic balance, and stress responses, leading to muscle functional decline. This highlights its indispensable role in maintaining skeletal muscle homeostasis. Furthermore, exercise—an effective intervention for improving muscle quality and function—may exert its beneficial effects through mechanisms closely linked to *CARM1* function. Therefore, this review systematically summarizes the roles of *CARM1* in skeletal muscle development, regeneration, material metabolism, and homeostasis based on its molecular structure and fundamental functions. It further explores *CARM1’s* functional manifestations in muscle atrophy and exercise adaptation, providing a theoretical framework for comprehensively understanding its pivotal role in physiological adaptation and muscle diseases, while evaluating its potential value as a therapeutic target.

## 1 Introduction

Skeletal muscle accounts for approximately 40%–50% of body weight and serves as the core tissue sustaining locomotor function and metabolic homeostasis. Its progressive decline in mass and function represents a common pathological feature of aging and various chronic diseases, including muscular dystrophy, cancer cachexia, and diabetic myopathy, severely impairing patients’ quality of life and increasing mortality rates (Sartori et al., 2021). The maintenance of skeletal muscle homeostasis relies on the regenerative potential of muscle stem cells, the differentiation efficiency of myoblasts, and the precise balance of multiple physiological processes, including protein synthesis and degradation, energy metabolism, and oxidative stress. The destruction of this balance leads to muscle atrophy and regeneration disorder, which is the direct cause of the above diseases (Bonaldo and Sandri, 2013). Therefore, an in-depth analysis of its regulatory mechanisms holds significant value in both physiological and clinical applications.

With the rapid advancement of epigenetics research, the central role of post-translational modifications in determining cellular fate and regulating function has become increasingly prominent. Among these, protein arginine methylation, as a key epigenetic mechanism, dynamically and reversibly modifies numerous substrate proteins, extensively participating in processes such as gene transcription, signal transduction, and cellular stress responses (Blanc and Richard, 2017). *Protein arginine methyltransferases (PRMTs)* are the key enzymes that catalyze this modification. According to the different types of methylation, *PRMTs* can be divided into type I (catalytic asymmetric dimethylation, ADMA), type II (catalytic symmetric dimethylation, SDMA), and type III (catalytic monomethylation, MMA) (Tewary et al., 2019). In recent years, the roles of *PRMTs* in metabolic tissues and the regulation of cell fate have garnered significant attention. They precisely regulate cellular proliferation, differentiation, metabolism, and apoptosis by methylating histones, transcription factors, chromatin remodeling complexes, and signaling molecules.

*Coactivator-Associated Arginine Methyltransferase 1 (CARM1)*, also known as *PRMT4*, is a member of this family. Transcriptome analysis revealed that the mRNA expression of CARM1 was significantly higher than that of other subtypes, such as *PRMT1* to *PRMT6*, in mouse skeletal muscle and C2C12 myoblast model; This expression spectrum has been strictly confirmed in human quadriceps femoris. The transcript abundance of *CARM1* is about 5 times higher than *Protein Arginine Methyltransferase 1(PRMT1), Protein Arginine Methyltransferase 5(PRMT5)*, and *Protein Arginine Methyltransferase 1(PRMT7)*, and 7-60 times higher than *Protein Arginine Methyltransferase 2(PRMT2)*, *Protein Arginine Methyltransferase 3(PRMT3)*, *Protein Arginine Methyltransferase 6(PRMT6)*, and *Protein Arginine Methyltransferase 9(PRMT9)*, occupying a dominant position (Wang et al., 2012; vanLieshout et al., 2019). This unique expression profile suggests *CARM1* may play a critical role in skeletal muscle biology. *CARM1* participates in key physiological processes such as cell fate determination, myofibrillar differentiation, and metabolic adaptation by catalyzing the arginine methylation of histones (e.g., Histone H3 Arginine 17, H3R17) and non-histone substrates (including various transcription regulators and signaling molecules) (Bauer et al., 2002; Jambhekar et al., 2019). Collectively, these findings demonstrate that *CARM1*-mediated protein methylation constitutes a pivotal hub linking epigenetic regulation to physiological and pathological states in skeletal muscle. However, current research remains fragmented, lacking systematic theoretical integration, which limits a comprehensive understanding of *CARM1’s* central role in skeletal muscle homeostasis.

Given that existing studies predominantly focus on isolated functional aspects of *CARM1* and lack systematic integration within the context of skeletal muscle, this review aims to establish, for the first time, a multifunctional regulatory network of *CARM1* in skeletal muscle. It delves into how *CARM1* precisely regulates skeletal muscle life activities at both the epigenetic and signaling levels through its methyltransferase activity, with particular emphasis on its regulatory mechanisms in muscular diseases and sex differences. It further explores the potential therapeutic applications of targeting *CARM1* for muscle-related disorders, aiming to provide a systematic theoretical foundation and research framework for deepening our understanding of skeletal muscle biology and developing novel therapeutic strategies.

## 2 Overview of *CARM1*

### 2.1 Structure of *CARM1*

*Coactivator-associated Arginine Methyltransferase 1 (CARM1)* belongs to the Type I PRMT family. Its name derives from its function as a nuclear receptor coactivator and its catalytic activity in asymmetric dimethylation of arginine residues (Chen et al., 1999). Molecularly, *CARM1* comprises approximately 608 amino acids with a molecular weight of about 63 kDa (Berger et al., 2011). Its structure consists of three segments: a central catalytic core domain (residues 150-470 in mouse *CARM1*), flanked by an N-terminal domain (residues 1–130) and a C-terminal domain (residues 480–608) (Teyssier et al., 2002). The catalytic core domain exhibits high sequence conservation across all PRMT family members and is the key region for its methylation function. The N-terminal and C-terminal domains are also crucial for *CARM1’s* transcriptional co-activation function, regulating *CARM1’s* activity through interactions with other proteins and other mechanisms (Teyssier et al., 2002).

### 2.2 Fundamental molecular functions of *CARM1*

As a type I arginine methyltransferase, *CARM1* primarily catalyzes asymmetric dimethylation at histone H3 position R17 (H3R17me2a) in skeletal muscle. During C2C12 cell differentiation, CARM1 modifies chromatin structure by transporting H3R17me2a—a critical histone mark—from the cytoplasm to the myonucleus, thereby driving muscle gene expression programs (Shen et al., 2018). Beyond histones, CARM1 recognizes multiple non-histone substrates, including the transcription coactivator *E1A Binding Protein p300/CREB-Binding Protein(p300/CBP)*, the RNA-binding protein *polyadenylate-binding protein 1 (PABP1)*, and *human antigen R (HuR)* (Li et al., 2002; Kaniskan et al., 2016). Among these, *HuR*, as a substrate, has been demonstrated to participate in regulating muscle differentiation processes, with its abnormalities linked to the pathogenesis of muscular dystrophy (Stone et al., 2021). The roles of other key substrates, such as the transcription coactivator *p300/CBP* and the RNA-binding protein *PABP1*, are primarily based on studies in non-muscle cell lines; their specific functions within skeletal muscle require further validation. In transcriptional regulation, *CARM1* mainly acts as a coactivator, enhancing gene transcription activity by methylating the H3R17 site (Jacques et al., 2016). Simultaneously, it regulates the activity and stability of transcription factors such as p160 family coactivators, p53, and NF-κB, precisely controlling the expression programs of specific genes (Chen et al., 1999). This provides crucial assurance for the precise regulation of skeletal muscle gene expression.

*CARM1’s* enzymatic activity undergoes multi-level fine-tuning, with its C-terminal self-methylation modifying catalytic efficiency (Kuhn et al., 2011). This enzymatic activity is crucial for cellular development, as demonstrated in mice deficient in *CARM1*. These mice exhibit phenotypes similar to those of gene knockout models, including embryonic lethality, impaired T cell development, defective adipocyte differentiation, and reduced transcriptional coactivation activity, underscoring the irreplaceable nature of its catalytic function (Kim et al., 2010). Notably, phosphorylation at position S595 serves as a key molecular switch for *CARM1’s* response to oxidative stress signals. This phosphorylation modification triggers *CARM1’s* transport from the nucleus to the cytoplasm, enabling it to target cytoplasmic substrates (Cho and Kim, 2024a). This localization shift confers *CARM1* a unique dual identity: as a nuclear transcription coactivator that regulates gene expression, and as a cytoplasmic stress-response enzyme that rapidly adjusts cellular homeostasis. This mechanism enables *CARM1* to couple epigenetic programs with transient cellular metabolic states tightly. These discoveries establish the molecular basis for understanding its complex role in the specific tissue of skeletal muscle.

However, current research on the specific functions of *CARM1’s* functional domains within skeletal muscle remains limited. Studies in other cell types indicate that its catalytic and regulatory domains are crucial for enzymatic activity. Yet, whether these structure-function relationships exhibit specificity in the skeletal muscle context, and how muscle-specific binding partners influence its function, remain critical questions to be addressed. Collectively, these regulatory mechanisms ensure that *CARM1* can respond to diverse physiological signals, exerting precise control over critical biological processes including skeletal muscle differentiation, metabolic regulation, and stress responses.

## 3 The role of *CARM1* in muscle development

### 3.1 The role of *CARM1* in regulating muscle stem cell activation and early differentiation

Satellite cells (i.e., muscle stem cells) reside beneath the basement membrane of muscle fibers and serve as the primary stem cells for skeletal muscle growth, repair, and regeneration (Mauro, 1961). They maintain low metabolic activity at rest but can rapidly activate, proliferate, and differentiate into mature muscle cells in response to stimuli such as muscle injury (Schultz et al., 1978; Bentzinger et al., 2012). The transcription factor *Paired Box 7 (PAX7)* serves as their specific marker and is crucial for maintaining stem cell properties and early differentiation. Mouse teratoma models demonstrate that *PAX7* deficiency severely impairs myogenic differentiation, obstructing myofibrillar formation and maturation (Florkowska et al., 2020). Its downstream target, *Myogenic Factor 5 (Myf5)*, plays a pivotal role in initiating differentiation, with its expression directly regulated by *PAX7* (McKinnell et al., 2008). In goat models, *Myf5* expression dynamics closely correlate with muscle maturation: it is broadly expressed early in development and progressively restricted to satellite cells and a few myonuclei as development progresses (Pani et al., 2025). *PAX7* and *Myf5* form a core axis regulating muscle stem cell fate, jointly governing maintenance and differentiation (Olguín and Pisconti, 2012). In late differentiation, *Myogenic Enhancer Factor 2C (MEF2C)* promotes the maturation of myogenic precursors into mature muscle fibers by activating muscle-specific genes (Piasecka et al., 2021).

**FIGURE 1:**
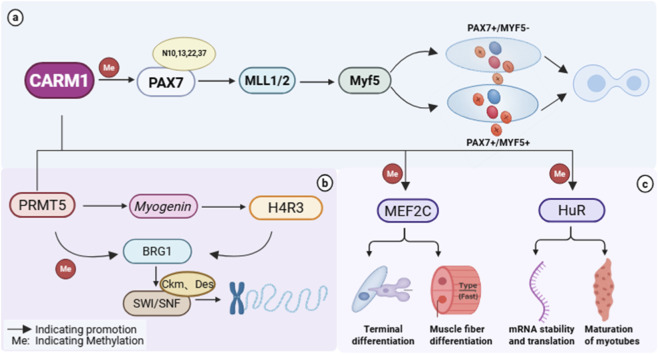
The role of *CARM1* in muscle development. **(a)** During early muscle stem cell differentiation, *CARM1* mediates specific methylation of the transcription factor *PAX7*. This modification creates binding sites for the MLL1/2 complex, activating *Myf5* transcription activity to balance stem cell self-renewal (*PAX7+/MYF5-*) and differentiation initiation (*PAX7+/MYF5+*), thereby regulating the critical asymmetric division of satellite cells; **(b)** During mid-to-late differentiation, *CARM1* synergizes with *PRMT5* to methylate *BRG1*, facilitating SWI/SNF complex recruitment to promoter regions of muscle-specific genes (e.g., Ckm, Des). This modulates chromatin accessibility, establishing the foundation for gene expression; **(c)** As differentiation progresses to late stages, *CARM*1 methylates *MEF2C* to promote muscle stem cell terminal differentiation and fast-twitch fiber differentiation. *CARM1* methylates HuR, affecting its stability and translation efficiency for multiple muscle differentiation-related mRNAs, thereby indirectly regulating myotube formation and maturation. Created in https://BioRender.com.

Current research on *CARM1* regulation mechanisms is primarily based on mouse and *in vitro* cell models. Kawabe et al. (Kawabe et al., 2012) demonstrated that *CARM1* specifically methylates N-terminal arginine residues (positions 10, 13, 22, 37) of *PAX7* during early differentiation, providing a binding site for the MLL1/2 complex. This recruits *histone 3, lysine 4 (H3K4)* methyltransferase to activate Myf5 transcription. This mechanism promotes the asymmetric division of satellite cells, balancing stem cell self-renewal (*PAX7+/MYF5-*) and differentiation initiation (*PAX7+/MYF5+*). In Duchenne muscular dystrophy (DMD) models, the inhibition of *CARM1* activity reduces satellite cell asymmetric division and impairs muscle regenerative capacity (Chang et al., 2018). This pathological phenomenon, conversely, validates the critical role of the *CARM1-PAX7-MYF5* regulatory axis in maintaining muscle stem cell pool homeostasis and governing initial differentiation. As differentiation progresses to later stages, *CARM1’s* regulatory focus shifts toward effectors like *MEF2C*. CARM1 directly interacts with the C-terminal region (amino acids 224–465) of *MEF2C*. In the presence of the SRC coactivator *Glutamate Receptor Interacting Protein 1 (GRIP-1)*, *CARM1* functions as a coactivator to enhance *MEF2C’s* transcriptional activation capacity, thereby activating muscle-specific gene expression and promoting myotube formation and terminal differentiation (Chen et al., 2002). This phased regulatory pattern, transitioning from early determinants to late effectors, enables *CARM1* to precisely control the entire process of myogenic differentiation precisely, ensuring the smooth progression of muscle development and regeneration programs (Figure 1).

It is worth noting that direct validation studies using human primary muscle cells and clinical samples are currently lacking. Although cross-species comparisons indicate the conservation of relevant transcription factors, the specific regulatory mechanisms of *CARM1* in human muscle stem cells require further experimental confirmation. This research gap limits our in-depth understanding of *CARM1’s* role in human muscle diseases.

### 3.2 Role of *CARM1* in regulating myoblast differentiation and maturation

Following stem cell activation and transition to myoblasts, *CARM1* continues to participate in regulating myoblast fusion and terminal differentiation into mature myotubes. This stage involves the temporal activation of multiple transcription factors and the precise regulation of muscle-specific gene expression networks.

*CARM1* exhibits a temporally specific synergistic interaction pattern with another PRMT family member, *PRMT5*, during muscle differentiation (Nie et al., 2018). These two enzymes catalyze distinct types of arginine methylation modifications: *PRMT5* primarily mediates symmetric dimethylation, while *CARM1* predominantly catalyzes asymmetric dimethylation, each of which is associated with specific gene regulatory functions (Xu and Richard, 2021). During early muscle differentiation, *PRMT5* is recruited to the promoter region of the myogenin gene. By modifying *histone H4 arginine 3 (H4R3)*, it promotes the binding of the ATP-dependent chromatin remodeling enzyme *Brahma-related gene 1 (BRG1)*, thereby restructuring chromatin and creating favorable conditions for the binding of myogenic transcription factors (Dacwag et al., 2007). As differentiation progresses into later stages, *CARM1* assumes an increasingly critical role. *CARM1* synergizes with *PRMT5* to methylate specific arginine residues of *BRG1*, thereby promoting the recruitment of the *SWItch/Sucrose NonFermentable (SWI/SNF)* chromatin remodeling complex to the promoter regions of muscle-specific genes (Ckm and Des), further regulating chromatin accessibility (Dacwag et al., 2009) (Figure 1). Experimental evidence indicates that *CARM1* deficiency significantly impairs this chromatin remodeling activity, leading to impaired expression of terminal differentiation genes, demonstrating that *CARM1* is essential for chromatin remodeling of late differentiation genes (Dacwag et al., 2009).

Beyond regulating chromatin states via epigenetic mechanisms, *CARM1* also participates in post-transcriptional regulatory networks of muscle differentiation by modifying RNA-binding proteins. *CARM1* specifically methylates *HuR*, precisely regulating its binding capacity, subcellular localization, and functional activity (Ravel-Chapuis et al., 2022) (Figure 1). This modification directly impacts *HuR’s* stability and translational efficiency for various muscle differentiation-related mRNAs containing AU-rich elements, thereby establishing a fine-tuned post-transcriptional regulatory mechanism that indirectly influences myotube formation and maturation.

During muscle fiber maturation, *CARM1* plays a further role in fiber type specialization and functional acquisition. In a zebrafish skeletal muscle development model, *CARM1* promotes the differentiation of fast-twitch fibers explicitly and influences the spatial localization of slow-twitch fibers by regulating *Myogenin* expression (Batut et al., 2011). *CARM1* deficiency causes impaired migration of slow-twitch fibers in zebrafish embryos, preventing them from reaching their correct anatomical positions. Mechanistic studies indicate that *CARM1* establishes a molecular environment conducive to specific fiber type differentiation by maintaining the expression balance among key transcription factors such as *Myogenin*, *Mef2C*, and *Myf5* (Batut et al., 2011). Further mouse studies revealed that *CARM1* selectively promotes the differentiation of type II fast-twitch fibers with minimal impact on type I slow-twitch fibers (Wang et al., 2012) (Figure 1). However, these findings are primarily based on zebrafish and rodent models, and whether identical regulatory mechanisms exist in large mammals (e.g., sheep, horses, pigs) and humans remains unclear. Given significant differences in myofiber composition, metabolic characteristics, and functional requirements across species, direct extrapolation of these findings to human clinical applications warrants caution. Future research should prioritize functional validation of *CARM1* in human skeletal muscle and clinically relevant large animal models.

Collectively, *CARM1* constructs a continuous, multi-tiered regulatory network spanning from stem cell activation to myofibrillar maturation during muscle development. This network exhibits three key characteristics: temporal specificity (cooperation with *PRMT5* at distinct developmental stages), multi-target regulation (controlling transcription factors, chromatin remodeling complexes, and RNA-binding proteins), and subtype selectivity (differentiated regulation of distinct myofibrillar types). Through these intricate regulatory mechanisms, *CARM1* participates in all stages of muscle development, ensuring precise coordination of muscle tissue formation and fully demonstrating the central role of epigenetic regulation in tissue development.

## 4 *CARM1* regulation of metabolic processes

### 4.1 Regulation of glucose metabolism

Glucose metabolism involves the absorption, storage, and breakdown of carbohydrates, achieving synthesis and degradation through a series of enzymatic reactions (Soo et al., 2023). In skeletal muscle, glucose metabolism not only supplies energy for muscle movement but also plays a crucial role in regulating insulin sensitivity, collectively supporting muscle health (Richter and Hargreaves, 2013). *CARM1* exhibits a perplexing dual role in regulating glucose metabolism.

In tumor and non-muscle cell models, *CARM1* typically functions as a direct modifier of metabolic enzymes, dynamically regulating enzyme activity through post-translational modifications, yet its effects demonstrate high heterogeneity. For instance, in tumor cells, *CARM1* methylates *Pyruvate Kinase M2 (PKM2)*. Methylated *PKM2* interacts with endoplasmic reticulum calcium channels IP3Rs (IP3R1/3) and inhibits their expression, thereby reducing mitochondrial calcium uptake, decreasing oxidative phosphorylation, and promoting aerobic glycolysis (Liu et al., 2017). Conversely, in fibroblasts, *CARM1*-mediated methylation at *PKM2-specific sites (R445/R447)* promotes tetramer formation, significantly enhancing enzyme activity (Abeywardana et al., 2018). This tissue-specific regulation is further validated in other metabolic enzymes. Moreover, in hepatocellular carcinoma and osteoblasts, *CARM1* induces complex metabolic reprogramming by methylating targets including *Malate Dehydrogenase 1 (MDH1)*, *Glyceraldehyde-3-Phosphate Dehydrogenase (GAPDH)*, and *Protein Phosphatase 1 Catalytic Subunit Alpha (PPP1CA)* (Zhong et al., 2018; Zhang et al., 2023). These studies indicate that in non-muscle tissues, *CARM1* primarily intervenes in the allosteric regulation of metabolic enzymes through extensive and variable protein methylation modifications.

However, in skeletal muscle, *CARM1* exhibits a distinctly high specificity. In skeletal muscle, *CARM1* appears to function primarily as a transcriptional coactivator, exerting precise control over the transcriptional network regulating glycogen metabolism. Loss-of-function studies reveal that among over 200 metabolism-related genes, *CARM1* deficiency specifically inhibits only core genes of the glycogen metabolic pathway—*Glycogen Synthase 1 (Gys1), Phosphoglycerate Mutase 2 (Pgam2)*, and *Muscle Glycogen Phosphorylase (Pygm)*, with no significant impact on genes involved in lipid oxidation or glucose homeostasis (Wang et al., 2012). This precise gene regulation function strictly depends on *CARM1’s* intrinsic enzymatic activity. This is demonstrated by the VLD mutant (SAM-binding domain mutation), which completely loses methylation activity and causes severe impairment of glycogen synthesis, while the E267Q mutant (catalytic site mutation), which partially retains activity, exhibits a dose-dependent intermediate phenotype (Wang et al., 2012). This finding reveals *CARM1’s* pivotal role as a central node in skeletal muscle glycogen homeostasis. Notably, the *CARM1*-regulated target gene network exhibits a high degree of overlap with human hereditary glycogen storage diseases (GSD). Loss-of-function mutations in *GYS1*, *PGAM2*, and *PYGM* genes are the direct causes of type 0 (Kollberg et al., 2007), X-linked (Zhang et al., 2022), and McArdle disease (Llavero et al., 2019)glycogen storage disorders, respectively. Furthermore, CARM1’s regulation of upstream signaling molecules, including *AMPK (α2/γ3)* and p38 Mitogen-Activated Protein Kinase (p38 MAPK), links its functional loss to pathological phenotypes such as Wolff-Parkinson-White syndrome (Wang et al., 2012). Thus, *CARM1* in skeletal muscle is not merely a simple enzyme-modifying factor but regulates skeletal muscle glycogen metabolism by integrating transcriptional programs.

In summary, *CARM1* exhibits complex and tissue-specific functions in regulating glycogen metabolism. Unlike its multifaceted, even contradictory “dual-sided” regulatory patterns in tumor and fibroblast cells, *CARM1* exhibits high specificity in skeletal muscle, precisely focusing its function on glycogen metabolism pathways. This establishes *CARM1’s* physiological and clinical significance as a core master regulator of skeletal muscle glycogen homeostasis. In-depth analysis of these mechanisms not only helps elucidate tissue-specific gene regulation patterns but also provides new theoretical foundations and potential therapeutic targets for the treatment of metabolic diseases.

### 4.2 Regulation of protein metabolism

At the cellular level, skeletal muscle structure is primarily determined by the dynamic equilibrium between protein synthesis and degradation within myocytes (Yin et al., 2021). However, under certain pathological conditions, when the rate of protein degradation exceeds its synthesis rate, skeletal muscle mass and volume significantly decline—a phenomenon termed muscle atrophy (Fisher et al., 2017). Research indicates that skeletal muscle atrophy is primarily associated with abnormal activation of the ubiquitin-proteasome system, with *CARM1* playing a crucial regulatory role in this process (Tintignac et al., 2015).

Muscle-specific E3 ubiquitin ligases Muscle Atrophy F-box (Atrogin-1) and *Muscle-specific RING Finger Protein 1 (MuRF1)* are essential components of this system. They specifically recognize and ubiquitinate myofibrils and muscle regulatory proteins, promoting their proteasomal degradation and thereby mediating muscle protein breakdown and myofibrillar atrophy (Pang et al., 2023). *CARM1* influences muscle mass by regulating its expression levels. Under muscle atrophy conditions, *CARM1* enhances the transcriptional activity of the core transcription factor *Forkhead box O3 (FOXO3)* by methylating specific arginine residues. In protein metabolism, this methylation primarily activates the ubiquitin-proteasome pathway (UPP), leading to upregulation of E3 ubiquitin ligases *Atrogin-1* and *MuRF1*. This accelerates myosin degradation, thereby promoting muscle atrophy (Liu et al., 2019). Another crucial function of the *CARM1-FOXO3* axis is regulating autophagy initiation, as detailed in Section 5.2 (Regulation of Autophagy in Skeletal Muscle). In muscle-specific *CARM1* knockout (mKO) mice, muscle mass loss following sciatic nerve denervation was significantly reduced, accompanied by decreased expression of downstream atrophy genes *MuRF1* and *Atrogin-1*, supporting *CARM1* as a pro-atrophic factor under these conditions (Liu et al., 2019). However, a comprehensive analysis of multiple studies suggests that *CARM1’s* role in muscle atrophy may be condition-dependent. Following short-term pharmacological inhibition of *CARM1* (e.g., using EZM2302), although its substrate methylation levels decreased, it failed to significantly mitigate the decline in muscle mass and muscle fiber cross-sectional area induced by neural disuse. It also did not reproduce the anti-atrophic effects observed in the aforementioned gene knockout model (Webb et al., 2023). This discrepancy suggests that *CARM1* function may possess redundancy or alternative compensatory mechanisms under acute or short-term suppression conditions. More importantly, *CARM1* gene and protein expression significantly increased in neurogenic disuse atrophy models, with sustained upregulation of intracellular localization and enhanced methylation activity. Conversely, in fasting-induced atrophy models, the methyltransferase activity of *CARM1* decreased in both mice and humans, while protein levels remained unchanged—a stark contrast indicating potentially distinct regulatory mechanisms for *CARM1* across different atrophy patterns (Stouth et al., 2024; Stouth et al., 2018). This condition-dependent expression pattern suggests *CARM1* may exert both pathological pro-atrophic effects and adaptive protective functions under certain circumstances. However, existing evidence has significant limitations. Temporal experimental analyses are needed to clarify whether *CARM1* upregulation is a driver or a consequence of muscle atrophy, and to reveal further whether it undergoes a dynamic transition between early protective and late pathological functions during disease progression.

Additionally, *CARM1* plays a crucial role in nonsense-mediated mRNA decay (NMD) (Sanchez et al., 2016). When β-globin mRNA containing a premature termination codon (PTC) (MT) and normal mRNA (WT) were separately transfected into MN-1 control cells and CARM1-knockdown cells, results showed significantly reduced degradation of PTC-containing mRNA in *CARM1*-knockdown cells. This finding confirms that *CARM1* promotes the degradation of mRNA containing premature termination codons (PTCs). Through this mechanism, *CARM1* assists cells in efficiently recognizing and clearing abnormal mRNAs containing PTC, thereby preventing their translation into potentially harmful incomplete proteins (Sanchez et al., 2016). In spinal muscular atrophy (SMA) models, CARM1 protein levels are significantly elevated in spinal motor neurons and muscle tissues. This leads to an abnormally enhanced NMD process, affecting the maintenance of muscle function and repair-related genes: *Growth Arrest and DNA Damage-inducible 45 alpha (Gadd45a)*, *Activity-regulated Cytoskeleton-associated protein (Arc)*, and *Asparagine Synthetase (Asns) mRNA*, further exacerbating muscle atrophy (Sanchez et al., 2016).

In summary, *CARM1* exhibits condition-dependent roles in protein metabolism regulation: under certain conditions (e.g., neurogenic disuse), it primarily promotes atrophy, while in other scenarios (e.g., fasting stress), it may exert adaptive regulatory functions. *CARM1* participates in muscle mass regulation by activating the ubiquitin-proteasome system through *FOXO3* methylation and promoting PTC degradation via nonsense-mediated mRNA decay (NMD). This complexity suggests that CARM1 may function both as a driver of atrophy and as a component of cellular stress responses. Its precise mechanisms of action and therapeutic target potential require further temporal and mechanistic studies to elucidate.

## 5 Role of *CARM1* in regulating skeletal muscle homeostasis

### 5.1 Regulation of the oxidative stress response

Oxidative stress represents a pathological state characterized by an imbalance between the production of reactive oxygen species (ROS) and the antioxidant defense system within cells. As a high-oxygen-consuming tissue, skeletal muscle is particularly susceptible to oxidative stress due to its dense mitochondrial network (Chen et al., 2022). As the most significant metabolic organ in the human body, skeletal muscle requires precise regulatory mechanisms to maintain oxidative equilibrium (Lian et al., 2022). Oxidative stress forms a characteristic vicious cycle: excessive ROS damages mitochondrial DNA and the electron transport chain, while damaged mitochondria produce more ROS, further diminishing muscle fiber contractility (Wen et al., 2025). Concurrently, sustained oxidative stress activates cellular senescence programs, leading to myocyte growth arrest and reduced regenerative capacity, thereby accelerating muscle functional decline (Duranti, 2023).

**FIGURE 2:**
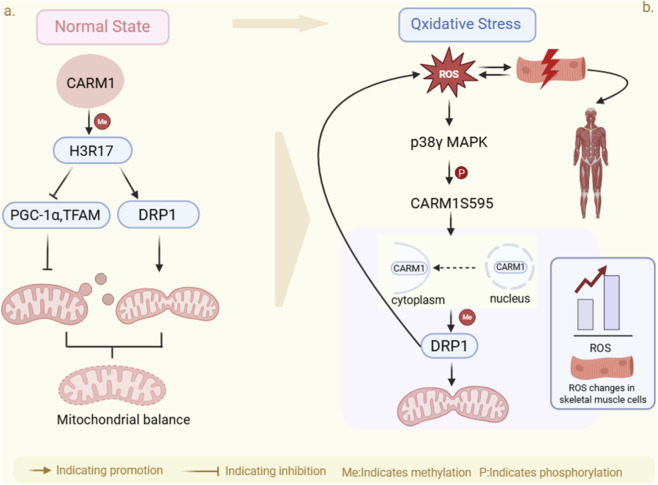
Molecular mechanism of *CARM1* regulating mitochondrial dynamic balance in different cellular states. **(a)** In the normal physiological state, *CARM1* plays a role in regulating mitochondrial biogenesis and fission. Through methylating H3R17, on one hand, *CARM1* suppresses mitochondrial biogenesis by inhibiting the mRNA and protein levels of *PGC-1α* and *TFAM*. On the other hand, it enhances the expression of *DRP1* protein to modulate mitochondrial fission, thereby preserving mitochondrial homeostasis. **(b)** Under oxidative stress conditions, *CARM1* induces mitochondrial dysfunction. Under oxidative stress conditions, elevated ROS levels activate p38γ MAPK, resulting in the phosphorylation of *CARM1* at the S595 site. This phenomenon promotes the translocation of *CARM1* from the nucleus to the cytoplasm, where it directly methylates *DRP1* to accelerate mitochondrial fission and form a positive feedback loop. Arrows indicate promotional effects, and T-shaped lines indicate inhibitory effects. Me: methylation; P: phosphorylation. Created in https://BioRender.com.

*CARM1*, an epigenetic regulator, plays a role in oxidative stress responses, primarily through transcriptional regulation and post-translational protein modification. Under basal conditions, *CARM1* is predominantly localized to the nucleus, precisely regulating key transcription factor networks to maintain mitochondrial homeostasis. Studies reveal that in *CARM1* knockout (KO) mouse embryonic fibroblasts (MEFs), expression of key mitochondrial biosynthetic regulators *Peroxisome Proliferator-Activated Receptor Gamma Coactivator 1-Alpha (PGC-α)* and *Mitochondrial Transcription Factor A (TFAM)* significantly increased, while expression of *Dynamin-Related Protein 1 (DRP1)*, which regulates mitochondrial fission, decreased. This led to a morphology characterized by elongated, fused mitochondria. Reintroduction of wild-type *CARM1* (WT) reverses this phenotype (Cho and Kim, 2024a). This demonstrates that *CARM1* operates in the nucleus by inhibiting *PGC-1α* and *TF*A*M* expression to limit mitochondrial biogenesis, while simultaneously promoting *DRP1* expression to regulate mitochondrial fission. This bidirectional regulatory mechanism plays a crucial role in maintaining the balance between mitochondrial number and function (Figure 2).

More critically, *CARM1* responds to oxidative stress signals by undergoing subcellular localization shifts, thereby establishing a precise positive feedback regulatory loop. Studies show that under oxidative stress conditions induced by Hydrogen Peroxide (H_2_O_2_) or Lipopolysaccharide (LPS), *CARM1* translocates from the nucleus to the cytoplasm in 10T1/2 cells (a mouse embryonic fibroblast cell line). A process blocked by the p38 MAPK inhibitor (BIRB 796) or nuclear export inhibitor (EZM2302), indicating dependence on p38γ MAPK activation and *CRM1*-mediated nuclear export mechanisms (Cho and Kim, 2024a; Cho and Kim, 2024b). Mechanistically, oxidative stress elevates intracellular reactive oxygen species (ROS) levels, activating p38γ MAPK, which phosphorylates *CARM1* at the S595 site and promotes its cytoplasmic translocation (Cho and Kim, 2024a). In the cytoplasm, *CARM1* directly methylates and activates *DRP1*, thereby enhancing mitochondrial fission and promoting further ROS production, which accelerates cellular senescence (Cho and Kim, 2024a; Cho and Kim, 2024b). Under oxidative stress, CARM1 knockout MEF cells exhibited significantly elevated mitochondrial membrane potential (MMP) and oxygen consumption rate (OCR), with mitochondria adopting a more elongated and fused morphology; In contrast, CARM1-WT MEF cells under identical conditions exhibited markedly decreased MMP and OCR, further confirming *CARM1’s* negative regulatory role in mitochondrial function (Cho and Kim, 2024b). This positive feedback loop mechanism—“ROS-*p38γ*-*CARM1*-*DRP1*-more ROS”—is particularly pronounced under physiological and pathological conditions such as intense exercise or aging. It rapidly amplifies initial oxidative stress signals, triggering adaptive or pathological cellular responses (Figure 2).

It is worth noting that the molecular details of the aforementioned feedback circuit are primarily based on data from fibroblasts. Although this intricate molecular chain has not been fully replicated in skeletal muscle *in vivo*, multiple lines of evidence strongly suggest its existence and functional relevance. In skeletal muscle undergoing chronic oxidative stress associated with aging, disuse, or neuromuscular diseases, persistent activation of the p38 MAPK pathway, elevated *DRP1* activity, mitochondrial fragmentation, and substantial ROS accumulation have been observed (Odeh et al., 2020; Favaro et al., 2019). These *in vivo* pathological phenotypes closely align with the aforementioned cellular pathways, suggesting that the *CARM1*-mediated pathway may serve as a critical bridge linking oxidative stress to skeletal muscle mitochondrial dysfunction.

In summary, *CARM1’s* significance lies in its role as a molecular hub integrating nuclear transcriptional programs with cytoplasmic rapid stress responses. The “ROS–*p38γ*–*CARM1*–*DRP1-more ROS*” pathway elucidated in fibroblasts provides a compelling candidate model for understanding the amplification mechanisms of oxidative stress in skeletal muscle. Validating this cellular model within skeletal muscle and exploring its muscle-specific regulatory mechanisms represent future research directions. This holds significant theoretical value and may also provide novel potential targets for developing targeted strategies against muscle atrophy and degenerative diseases.

### 5.2 Regulation of Autophagy in Skeletal Muscle

Autophagy is a highly conserved intracellular degradation and recycling mechanism that selectively degrades damaged organelles, misfolded protein aggregates, and other cellular components via the lysosomal system, playing a central role in maintaining intracellular homeostasis (Levine and Kroemer, 2019). In skeletal muscle, precise regulation of autophagic flux is crucial for maintaining myofibrillar quality, repairing injuries, and balancing energy metabolism (Singh et al., 2022). *CARM1* establishes a sophisticated multi-tiered regulatory network through arginine methylation, coordinating three key pathways to maintain muscle autophagy homeostasis jointly.

During the early stages of autophagy initiation, *CARM1* controls the activation of *AMP-activated protein kinase (AMPK)*, the central energy sensor, through epigenetic mechanisms. *AMPK* serves as a core regulator of cellular energy metabolism, activating autophagy in response to energy stress to maintain energy balance (Feng et al., 2024). Stouth et al. (Stouth et al., 2020)revealed that *CARM1*-mediated methylation is a prerequisite for *AMPK* activation. Under neurogenic muscular atrophy and energy stress conditions, the interaction between *CARM1* and *AMPK* significantly intensifies, catalyzing ADMA methylation at the 5th position of *AMPK*. This methylation event is crucial as it provides the molecular basis for *AMPK Thr172* phosphorylation and subsequent kinase activation. Mechanistic studies indicate that *CARM1*-activated *AMPK* precisely initiates autophagy through a dual mechanism: on one hand, *AMPK* directly phosphorylates the Ser555 activation site of *Unc-51-like autophagy activating kinase 1 (ULK1)*; Second, it suppresses *Mechanistic Target of Rapamycin (mTOR)* complex activity, thereby releasing *mTOR’s* inhibitory phosphorylation of ULK1 Ser757 (Stouth et al., 2020; Park et al., 2023). However, in skeletal muscle-specific *CARM1* knockout (mKO) mice, impaired *AMPK* methylation prevents its phosphorylation-mediated activation, triggering a cascade of events: Ser555 phosphorylation of *ULK1* fails to increase under stress conditions, and *mTOR* inhibition of Ser757 cannot be relieved, resulting in complete disruption of the autophagy initiation signal (Stouth et al., 2020). Fasting experiments further confirmed that *CARM1* deficiency reduces the conversion of lipidated *LC3 (LC3-II)* and impairs autophagy flux (Stouth et al., 2024). This indicates *CARM1* is a critical component in skeletal muscle’s response to energy stress and initiation of the autophagy program.

Following rapid initiation, *CARM1* further maintains and amplifies autophagy efficacy by co-activating the *FOXO3* transcription factor. Unlike the acute stress response mediated by *AMPK*, *FOXO3* primarily governs the transcriptional expression of autophagy-related genes (ATGs) (Mammucari et al., 2007). *CARM1* significantly enhances *FOXO3’s* nuclear transcriptional activity through asymmetric dimethylation (ADMA) modification, specifically upregulating autophagy-initiating factors *Autophagy-related protein 13 (Atg13)* and *Autophagy-related protein 14 (Atg14),* thereby promoting sustained autophagosome formation (Liu et al., 2019). Notably, this mechanism aligns with CARM1’s regulation of the ubiquitin-proteasome system (see Section 4.2), which jointly drives muscle atrophy. Functional validation revealed the critical physiological significance of this regulation: In both neurotomy and dexamethasone-induced muscle atrophy models, *CARM1* knockdown or inhibition significantly slowed atrophy progression, manifested as increased wet weight of the tibialis anterior muscle, markedly improved muscle fiber cross-sectional area, reduced methylation levels of *FoxO3*, and decreased *LC3-II/LC3-I* ratio—a marker of autophagy (Liu et al., 2019). This finding not only elucidates the molecular mechanism by which *CARM1* coordinates autophagy and protein degradation at the transcriptional level but also suggests its potential as a therapeutic target for muscle atrophy.

As the final line of defense in quality control, mitochondria are crucial for maintaining muscle energy homeostasis, and their dysfunction is a core feature of muscle aging and disease (Fealy et al., 2021). *CARM1* resides upstream of the classical *PTEN-induced putative kinase 1-Parkin (PINK1-Parkin)* pathway. It enhances the ubiquitinating activity of the E3 ubiquitin ligase Parkin by directly methylating it, ensuring damaged mitochondria are promptly tagged and cleared. Under fasting conditions, *CARM1* deficiency disrupts Parkin-mediated clearance mechanisms, resulting in the abnormal accumulation of dysfunctional mitochondria and a decrease in oxidative phosphorylation complex activity (Stouth et al., 2024). Clinical sample analysis reveals that the dynamic changes in *CARM1* activity in human muscle during fasting highly correlate with mouse models (Stouth et al., 2024). This suggests that *CARM1* functions as a crucial epigenetic regulator, coordinating mitochondrial quality control under nutritional stress.

In summary, *CARM1* establishes a multi-tiered autophagy regulatory network in skeletal muscle by synergistically modulating three key pathways: the *AMPK-mTOR* signaling pathway, *FOXO3*-mediated autophagy gene transcription, and Parkin-dependent mitophagy. This suggests that dysregulated *CARM1* expression correlates with muscle diseases, and targeting CARM1 may offer a novel therapeutic strategy for muscle atrophy. However, how *CARM1* dynamically balances pathway activities under different physiological states and whether tissue-specific co-regulators exist to recognize potential tissue-specific co-factors remain challenges for future research.

## 6 CARM1 and exercise

Skeletal muscle serves as the core motor organ of the human body, with its intricate functions and adaptability forming the cornerstone for maintaining health and optimizing athletic performance (Lim et al., 2022). Exercise stimuli induce a series of complex physiological and molecular changes in skeletal muscle, including myofibrillar type switching, mitochondrial proliferation, and metabolic reprogramming (Spaulding and Selsby, 2018). *CARM1* plays a pivotal role in this process, with its expression and activity precisely regulated by exercise timing. Transcriptomic and proteomic analyses have confirmed *CARM1* as one of the most highly expressed members of the *PRMT* family in human quadriceps femoris muscle, suggesting its critical hub status in skeletal muscle physiological regulation (vanLieshout et al., 2019).

Exercise stimulation induces time-dependent changes in *CARM1* expression and activity. During the acute exercise response phase (90-min treadmill running), H3R17 methylation status significantly increased in mouse gastrocnemius myonuclei, accompanied by elevated *CARM1* methyltransferase activity in the myonucleolar compartment (Vanlieshout et al., 2018). This suggests *CARM1*-mediated histone methylation serves as an early initiation signal for skeletal muscle adaptive remodeling. In a chronic exercise adaptation model, skeletal muscle-specific *CARM1* knockout (mKO) mice exhibited significantly reduced exercise endurance and reduced mitochondrial volume after 8 weeks of voluntary wheel running, consistent with findings of sustained *CARM1* protein upregulation in humans following 2 weeks of Sprint Interval Training (SIT) (vanLieshout et al., 2019; Vanlieshout et al., 2024). This confirms the species conservation of *CARM1* expression changes during exercise adaptation, where it mediates the link between the exercise stimulus and skeletal muscle adaptive remodeling through regulation of expression and subcellular relocalization.

*CARM1* exhibits sex-specific regulation during skeletal muscle exercise, with effects that depend on the exercise pattern. In a single acute running test, male skeletal muscle-specific *CARM1* knockout (mKO) mice showed impaired exercise endurance accompanied by reduced expression of the key metabolic regulator *PGC-1α* (vanLieshout et al., 2022). However, in an 8-week chronic voluntary wheel-running experiment, female *CARM1* mKO mice exhibited a sharp decline of approximately 70% in daily running distance, whereas male mKO mice showed no significant difference (Vanlieshout et al., 2024). This result eliminated the commonly observed female exercise advantage, aligning the exercise capacity of male and female mKO mice. This sex difference suggests *CARM1* may play a critical role in exercise motivation or fatigue tolerance in female mice, potentially through estrogen signaling pathways. The exercise endurance advantage in females is partially dependent on estrogen regulation, which modulates skeletal muscle function through receptors such as ERα and ERRα (Oydanich et al., 2019; Yoh et al., 2023). As a coactivator of ER-mediated transcription, *CARM1* promotes the expression of estrogen-responsive genes by methylating histones H3 at Arg17 and Arg26 sites or directly methylating ERα and its cofactors (Habashy et al., 2013; Peng et al., 2020). Consequently, its absence may disrupt this signaling pathway and impair exercise performance. However, despite histological analyses supporting these associations, *in vivo* evidence remains lacking for direct binding or methylation modifications between *CARM1* and ER or its downstream nuclear receptors (e.g., ERRα) within skeletal muscle cell nuclei via ChIP/Co-IP assays. Consequently, future studies should focus on elucidating how *CARM1* specifically influences metabolic reprogramming in female muscle. Additionally, we found that basal *CARM1* expression levels in female muscle tissue are on average 25% lower than in males (Webb et al., 2023). This lower baseline expression may amplify the relative loss of activity caused by *CARM1 d*eficiency, increasing female muscle dependence on estrogen-*CARM1*-mediated expression of energy metabolism genes like *PGC-1α* and *AMPK*. This explains the more severe decline in endurance observed in female mKO mice (Park et al., 2023; Yadav et al., 2003).

The mechanism of *CARM1 a*ction in skeletal muscle is highly complex and involves synergistic interactions within the *PRMT* family. In the absence of *CARM1*, *PRMT7)*expression is significantly upregulated, potentially serving as a compensatory mechanism to maintain skeletal muscle function (Wei et al., 2021; vanLieshout and Ljubicic, 2019). Concurrently, *PRMT1* and *PRMT5* also participate in metabolic regulation, forming a functionally complementary network with *CARM1* (vanLieshout and Ljubicic, 2019).

In summary, *CARM1* plays a crucial role in skeletal muscle exercise adaptation, with its function being comprehensively regulated by exercise patterns, muscle type, and sex differences. These findings not only provide a novel epigenetic perspective for understanding sex differences in skeletal muscle exercise-induced remodeling but also emphasize the importance of fully considering muscle type specificity and sex factors in future research. Future studies should prioritize validating the direct interaction between *CARM1* and the estrogen receptor using techniques such as ChIP/Co-IP, thereby providing a more robust molecular foundation for developing gender-specific exercise intervention strategies.

## 7 Discussion

This review systematically outlines the pivotal regulatory role of *CARM1* as a spermidyl methyltransferase in skeletal muscle physiology. *CARM1* exhibits multi-level, multi-stage regulatory functions in skeletal muscle development, metabolism, and the maintenance of homeostasis. From the activation of muscle stem cells and their asymmetric division, to myoblast differentiation and myofibrillar type specialization, and further to metabolic regulation and stress responses, *CARM1* precisely modulates key transcription factors, chromatin remodeling complexes, metabolic enzymes, and autophagy-related proteins through its methyltransferase activity, thereby constructing a complex regulatory network. In metabolism, *CARM1* specifically regulates the expression of key genes involved in glycogen metabolism, with its dysregulation linked to glycogen storage diseases. In protein metabolism, *CARM1* plays a role in the onset and progression of muscle atrophy by regulating the ubiquitin-proteasome system and nonsense-mediated mRNA decay. In regulating oxidative stress and autophagy, *CARM1* influences mitochondrial function and autophagy flux through the “ROS–p3*8γ*–*CARM1*–*DRP1*” positive feedback loop and multiple pathways, including the *AMPK-mTOR*, *FOXO3*, and *Parkin* pathways, thereby regulating muscle homeostasis and aging processes. Furthermore, *CARM1* exhibits time- and sex-dependent regulation during exercise adaptation, with its expression and activity modulated by exercise pattern, intensity, and sex, suggesting its essential and complex role in exercise physiology and metabolic health.

Despite these advances, several key challenges remain in understanding *CARM1’s* role in skeletal muscle physiology and pathology: (1) The precise mechanism underlying *CARM1* substrate specificity remains incompletely elucidated, particularly how it selectively targets different substrates under varying physiological conditions; (2) The spatiotemporal specificity of *CARM1* regulatory networks and their differential actions across distinct muscle fiber types require further investigation; (3) How *CARM1*-mediated epigenetic memory influences long-term skeletal muscle adaptation and disease progression remains unclear. Future research should focus on the following directions: First, apply single-cell epigenomics technologies to decipher the precise role of *CARM1* in muscle stem cell fate determination; second, develop *CARM1* substrate-specific inhibitors to explore their potential for targeted intervention in diseases such as muscular atrophy and diabetic myopathy; Third, integrating a gender medicine perspective to investigate the differential mechanisms of *CARM1*-mediated epigenetic regulation in skeletal muscle adaptation across genders. Finally, establishing an integrated bioinformatics platform to construct a multi-level regulatory network model of *CARM1* in skeletal muscle.

In summary, as a pivotal hub linking epigenetic modifications to skeletal muscle function, *CARM1* not only offers new insights into muscle physiology and pathology but also provides potential targets for developing personalized, precision intervention strategies for skeletal muscle disorders. As research progresses, the full landscape of *CARM1’s* regulatory network will gradually emerge, paving new pathways for maintaining skeletal muscle function and intervening in related diseases.
